# Diagnosis of a public policy: an introduction to user fee exemptions for healthcare in the Sahel

**DOI:** 10.1186/1472-6963-15-S3-S2

**Published:** 2015-11-06

**Authors:** Jean-Pierre Olivier de Sardan, Valéry Ridde

**Affiliations:** 1 Laboratoire d'Etudes et de Recherche sur les Dynamiques Sociales et le Développement Local (LASDEL), BP 12901, Niamey, Niger; 2 École des Hautes Etudes en Sciences Sociales (EHESS), France; 3 Department of Social and Preventive Medicine, School of Public Health, University of Montreal, Montreal, Québec, Canada; 4 Research Centre of the University of Montreal Hospital Centre (CRCHUM), Montreal, Québec, Canada

## Abstract

During the last ten years, Burkina Faso, Mali and Niger have opted for selective user fee exemption policies, while remaining within the general framework of cost recovery. But they have each developed their own particular institutional mechanisms, different from those of their neighbour. This was the topic of a comparative research program combining both quantitative and qualitative surveys over a four-year period. This special issue presents papers setting exemption policies in the wider context of public policy and the day-to-day functioning of health systems (part 1); presenting overarching case studies (part 2); and reflecting on our methodological approach (part 3).

User fee exemption policies were introduced in Burkina Faso, Mali and Niger during the first decade of this century. They cover several sector-based measures ('free healthcare' in everyday language), and sometimes come on top of high levels of subsidies which enabled significant reductions in the cost of certain drugs and treatments.

From the late 1980s, these three countries were - and still are - subject to a comprehensive system of cost recovery at the point of delivery (a policy introduced following the Bamako Initiative), or, to be more precise, a system of partial payment of drugs and services by the user. Only a small proportion of the costs are actually recovered as the amounts charged to the users do not take salaries, investments or recurrent costs, which are all paid by the state, into account, and represent only a small percentage of the overall health budget (an order of magnitude of five percent is often cited at state level [1,2]. Nevertheless, the sums recovered by health centres enabled them to buy drugs and cover certain local expenses.

However, for public health reasons, cost recovery has always been subject to a variety of sector-based exceptions, determined by the nature of the disease or intervention involved. For example, mass immunization (National Immunization Days) and routine vaccinations as part of the Extended Programme of Immunization (EPI), treatment relating to tuberculosis, leprosy, noma and Guinea worm, and measures for the prevention of epidemics all remained free of charge for users. The Bamako Initiative also made provision for a system that waived payment for patients who were too poor to pay for their treatment, however this system has never really been implemented (with regard to Burkina Faso, cf. [3]; for other countries in the region, see [4]).

This exclusion of the most vulnerable and the low health indicators in Africa, which are jeopardizing the achievement of the Millennium Development Goals (MDGs), explain the many criticisms of cost recovery that have mounted up within the NGOs, the research community and international organizations since the 1990s (cf. Ridde, this issue). This growing pressure for the abolition of the financial barriers to healthcare is clearly positioned within the progressive trend towards universal coverage. An international consensus has set itself the goal of ensuring that, by 2030, all populations, regardless of earnings, geographical location and gender, benefit from the coverage of 80% of basic health services, and 100% protection against the financial risks associated with direct payment [5].

This context explains why - over and above the three countries considered here and at around the same time - sector-based exemption policies were developed and implemented in a number of countries in Africa from the early years of this century [6].

## Why these three countries?

From a comparative perspective, the choice of Burkina Faso, Mali and Niger can be justified first and foremost because they are Francophone countries which, compared to English-speaking countries, have received little attention with regard to health funding, in general, and user fee exemption, in particular.

In addition, these countries are neighbours and despite considerable disparities in terms of medical and paramedical personnel (Burkina Faso has 0.5 doctors and 5.7 nurses and midwives per 10,000 inhabitants; Mali has 0.8 and 4.3, respectively; and Niger 0.2 and 1.4), share the same political, economic, social and health context for the most part. From a comparative point of view, in particular, they offered a specific advantage. On the one hand, all three have opted for sector-based user fee exemption policies (either in full or in part), while remaining within the general framework of cost recovery. In doing so, they highlight other, more ambitious policies pursued in other countries, like Ghana, Tanzania and South Africa, which rely systematically on income tax, VAT and health insurance [7,8]. On the other hand, they have each developed their own particular institutional mechanisms for user fee exemption, which are markedly different from those of the two other countries.

It will be seen that we have consciously opted for a *comparativism based on proximity*, which refers to contexts that are closely related and similar. This kind of comparativism appears more fruitful (and less common) to us than the frequent attempts made by epidemiology and public health studies to undertake large-scale comparisons, both within and between continents, which take no account of the profound cultural, political and historical differences between the countries being compared.

Another reason for choosing these countries was the presence of competent researchers on the ground with a thorough knowledge of how the health systems actually work in practice (something that differs considerably from their official functioning). In the case of investigations of an anthropological nature, in particular, this mastery of contexts and research areas is a decisive factor. The existence of a laboratory in Niger with an international reputation in this field (LASDEL, http://www.lasdel.net), which acted as the pivotal institution in the research programme and had already entered into collaborative ventures with both Mali and Burkina Faso as well as with the University of Montreal, was also an important asset.

## The research programme, its results and this 'special issue'

The articles in this supplement present some reflections based on a research programme entitled "*The abolition of payment for healthcare in West Africa (Mali, Burkina Faso and Niger)*", which was carried out in 2008 and led to a publication in French [9]. At that time, studies on these exemptions and their effects were thin on the ground [10]. Since then, however, such projects have become far more numerous, especially in English-speaking Africa [11]. These publications all testify to a significant increase in access to healthcare among the populations targeted by this 'free treatment'. Apart from a few exceptions [7,12,13], however, they have paid far less attention to other dimensions, such as the quality of care, actors' perceptions, institutional and organizational rationales and the strategies adopted by health workers.

Studies combining both quantitative and qualitative surveys were undertaken over a four-year period in Burkina Faso, Mali and Niger and involved a number of researchers from these countries, but also from Canada, under the coordination of J. P. Olivier de Sardan (LASDEL, Niger) and V. Ridde (University of Montreal, Canada). The funding was provided jointly by the IDRC (Canada) and the French Development Agency (ADF).

Various findings from this research programme are already available in English [14] and in French [9]. We may summarize the most relevant ones. The decision making process was characterized by a paradoxical combination of presidential voluntarism and external pressure, a lack of technical and financial preparation and a poor communication towards users as well as healthcare workers. The implementation process was characterized by insufficient funding (which turned to be dramatic in the case of Niger), shortages and delays, bureaucratic complexity, fuzzy edges of free healthcare, inconsistent half measures, a latent opposition of a majority of healthcare workers, and no proper monitoring. Concerning the effects, the intended effect of an increase in the numbers of health center visits by removing financial barrier was achieved. But we have identified many unexpected effects, the most important ones being a decline of quality of healthcare deliverance and widespread tinkering on the side of healthcare providers in order to keep the system running or to preserve personal advantages and informal practices.

For this special issue, we have given priority to other dimensions, hitherto unpublished in English, by setting exemption policies in the wider context of public policy and the day-to-day functioning of health systems (Part 1); by presenting overarching case studies (Part 2); and by reflecting on our methodological approach (Part 3).

1. Olivier de Sardan & Ridde (this issue) provide an overview of what has been achieved by the work done on public health policies and health systems, and of how these two strands have recently been brought together in a relatively new combination, i.e. health policy and systems research (HPSR) [15-17], an approach broadly in tune with our own research. However, this connection has not always been an obvious one, and has certainly not been taken on board everywhere, especially as far as West Africa is concerned, where studies linking the two dimensions are few and far between. Moreover, public policy on health matters should not be related to health systems alone (in a perspective confined to public health) but also to public policies in general, of which they constitute a subset (in a perspective offering an "public policy analysis"). By the same token, health systems gain from the occasional comparison with other public services, which, again, is something that rarely happens. Indeed, despite their obvious specificity, they are a subset of the services delivered by the method of bureaucratic governance [18,19]. Why dialogues between health policies and public policies or between health systems and public services are so thin on the ground might be due to a number of reasons: the way in which research programmes are organized, the difficulty of conducting interdisciplinary investigations, the division of labour between academic fields, the specialized nature of scientific publications and the way they operate etc. And yet, the analysis Ridde offers (in this issue) of the rapid succession involving these two, virtually antagonistic public health policies of cost recovery and user fee exemptions reveals many inconsistencies that are common to health policies and public policies, and testifies to just how difficult it is for research outcomes to influence the emergence and content of public policies, in the area of health as in everything else.

2. However, these inconsistencies are particularly acute in the case of user fee exemption policies. In the three countries considered, when implemented, these policies have fallen foul of the negative effects of the malfunctions of health systems: insufficient funding, inadequate control of inputs, ineffectual management of human resources, poor communication and a deterioration in relations with patients (for an analysis of some of these malfunctions based on anthropological investigations carried out in West Africa [20], particularly in Mali and Niger, as shown by Touré, and Diarra and Ousseini, respectively (this issue). Exemption policies have sometimes highlighted these malfunctions, and have sometimes made them worse. Consequently, the results give a mixed picture. On the one hand, an indisputable rise in attendances at health centres happened, in the case of under-fives with malaria (albeit a lower increase than expected). On the other hand, both Touré and Diarra and Ousseini bear witness to the countless problems encountered on a daily basis by both health workers and users in the implementation of exemptions. They reveal the existence of a real threat, perceived by all stakeholders and the research team to the quality of healthcare when there is no longer an NGO in support to compensate for the shortcomings of states. It is therefore legitimate to raise the question regarding: "quantity without quality? (...) Giving priority to quantity over quality in the name of access for the poorest: this is the very policy that was pursued by the World Bank in the area of education, and which did so much harm to the education systems in the three countries under consideration. It is vital that health should not follow this deplorable example!" [21]. In contrast, when international NGOs are present, quality is delivered. But then the central problem arises as to what happens once this temporary lifeline is severed. Olivier de Sardan et al. (this issue) describe the experiences of three NGOs that have supported user fee exemptions and question the capacity of the health systems to sustain these experiments after the NGOs have left, and, even more so, the capacity to 'scale them up'. The conclusion is obvious: every public policy should also involve, at the same time, a concerted effort to reform the health system, an effort that casts the net more widely by taking the national picture, the actual capacities of the state and the realities of day-by-day delivery of public services on the ground into account.

3. To obtain the outcomes presented here, it was absolutely vital to adopt methods used in anthropology to complement the quantitative methods more usually adopted in the area of public health (which were also represented in our programme). Hence, Ridde & Olivier de Sardan (this issue) report on this experiment which combines an ethnographic and an epidemiological approach and qualitative and quantitative methods. Despite recent progress in the area of health, where mixed methods are gaining ground [22], these combinations are more often prescribed than acted on, let alone analysed in the existing literature. They do, however, pose complex problems. How can a common problematic be jointly processed by scientists from different disciplines? How can each of the two methodological approaches ask questions, to which the other must attempt to reply? How are the two approaches to be combined in the interpretation of data? How can the domination of one approach by the other be avoided? How is the rigour of the qualitative and quantitative procedures to be combined when they do not yield the same kind of evidence? Moreover, although the quantitative method is familiar to decision-makers in public health, the qualitative method (which is characteristic of ethnography) unsettles them, all the more so because it 'lays its cards on the table', often giving expression to the kind of criticism more frequently associated with the free flow of private conversations than with the more guarded comments made in public. Consequently, Olivier de Sardan (this issue) describes the various misunderstandings that arose during the investigations between the research team and the health system managers, and the nature of their responses.

## Competing interests

None

## Authors' contributions

JPOS and VR conceived the idea, wrote the draft and final version of the manuscript. All authors read and approved the final manuscript.

## Declarations

This article has been modified from the chapters **Introduction. Diagnostic d'une politique publique: les exemptions de paiement pour les soins de santé au Sahel** by J.-P. Olivier de Sardan and V. Ridde [23], **Les spécificités des politiques publiques et des systèmes de santé en Afrique sahélienne** by J.-P. Olivier de Sardan and V. Ridde [24], and **La quantité sans la qualité? Mises en forme et mises en oeuvre des politiques d'exemptions de paiements au Sahel** by J.-P. Olivier de Sardan [25] in the book *Une politique publique de santé et ses contradictions. La gratuité des soins au Burkina Faso, au Mali et au Niger*, J.-P. Olivier de Sardan and V. Ridde (eds), 2014, Karthala, Paris, with the permission of the publisher. The publication of this supplement was funded by a grant from International Development Research Centre (IDRC), Ottawa, Canada.

This article has been published as part of *BMC Health Services *Research Volume 15 Supplement 3, 2015: User Fee Exemption Policies. The full contents of the supplement are available online at http://www.biomedcentral.com/bmchealthservres/supplements/15/S3.

## References

[B1] Nolan B Turbat V Cost Recovery in Public Health Services in Sub-Saharian Africa 1995 Washington, DC: World Bank [Bank W (Series editor): EDI Technical Materials]

[B2] Gilson L The lessons of user fee experience in Africa Health Policy Plan 1997 12 4 273 285 10176263 10.1093/oxfordjournals.heapol.a018882

[B3] Ridde V "The problem of the worst-off is dealt with after all other issues": the equity and health policy implementation gap in Burkina Faso Soc Sci Med 2008 66 6 1368 1378 18248864 10.1016/j.socscimed.2007.10.026

[B4] Gilson L Kalyalya D Kuchler F Lake S Organa H Ouendo M The equity impacts of community financing activities in three African countries Int J Health Plann Manage 2000 15 4 291 317 11246899 10.1002/hpm.599

[B5] World Bank La Couverture Universelle en santé: Suivi des progrès Aà l'échelon national et mondial 2014 Cadre, mesures et objectifs. Geneva: WHO, World Bank

[B6] Robert E Samb O Pour une cartographie des soins de santé gratuits en Afrique de l'Ouest Afr Contemp 2012 243 100 102

[B7] Macha J Harris B Garshong B Ataguba JE Akazili J Kuwawenaruwa A Borghi J Factors influencing the burden of health care financing and the distribution of health care benefits in Ghana, Tanzania and South Africa Health Policy Plan 2012 27 suppl 1 i46 i54 22388500 10.1093/heapol/czs024

[B8] Gilson L Erasmus E Borghi J Macha J Kamuzora P Mtei G Using stakeholder analysis to support moves towards universal coverage: lessons from the SHIELD project Health Policy Plan 2012 27 suppl 1 i64 i76 22388502 10.1093/heapol/czs007

[B9] Olivier de Sardan J-P Ridde V Une politique publique de santé et ses contradictions. La gratuité des soins au Burkina Faso, au Mali et au Niger 2014 Paris: Karthala

[B10] Ridde V Morestin F Belaid L Sanni Y. Ottawa Politiques contemporaines de gratuité des soins en Afrique Maux Choses Santé Acteurs Pratiques. Systèmes de Santé dans le Tiers-Monde 2010 Presses de l'Université d'Ottawa 207 242

[B11] Meessen B Hercot D Noirhomme M Ridde V Tibouti A Tashobya CK Gilson L Removing user fees in the health sector: a review of policy processes in six sub-Saharan African countries Health Policy Plan 2011 26 suppl 2 ii16 ii29 22027916 10.1093/heapol/czr062

[B12] Walker L Gilson L "We are bitter but we are satisfied": nurses as street-level bureaucrats in South Africa Soc Sci Med 2004 59 6 1251 1261 15210096 10.1016/j.socscimed.2003.12.020

[B13] Gilson L McIntyre D Removing user fees for primary care in Africa: the need for careful action 2005 331 7519 762 765 10.1136/bmj.331.7519.762 PMC1239983 16195296

[B14] Ridde V Olivier de Sardan J-P Zerah D, A Savoir Abolishing User Fees for Patients in West Africa: Lessons for Public Policy 2013 Paris: AFD, Paris http://recherche.afd.fr

[B15] Bennett S Agyepong I Sheikh K Hanson K Ssengooba F Gilson L Building the Field of Health Policy and Systems Research: An Agenda for Action PLoS Med 2011 8 8 e1001081. 21918641 10.1371/journal.pmed.1001081 PMC3168867

[B16] Gilson L Hanson K Sheikh K Agyepong IA Ssengooba F Bennett S Building the field of health policy and systems research: social science matters PLoS Med 2011 8 8 e1001079. 21886488 10.1371/journal.pmed.1001079 PMC3160340

[B17] Sheikh K George A Gilson L People-centred science: strengthening the practice of health policy and systems research Health Res Policy Syst 2014 12 19 24739525 10.1186/1478-4505-12-19 PMC4018943

[B18] Olivier de Sardan J-P The Eight Modes of Local Governance in West Africa IDS Bull 2011 42 2 22 31

[B19] Olivier de Sardan J-P The Delivery State in Africa Interface Bureaucrats, Professional Cultures and the Bureaucratic Mode of Governance States at Work 2014 Leiden: Bierschenk Thomas & Olivier de Sardan Jean-Pierre

[B20] Jaffré Y Olivier de Sardan J-P Une médecine inhospitalière Les difficiles relations entre soignants et soignés dans cinq capitales d'Afrique de l'Ouest 2003 Paris: APAD, Karthala

[B21] Olivier de Sardan J-P Olivier de Sardan JP, Ridde V La quantité sans la qualité? Mises en forme et mises en œuvre des politiques d'exemption de paiement au Sahel Une politique publique de santé et ses contradictions La gratuité des soins au Burkina Faso, au Mali et au Niger 2014 Paris: Khartala

[B22] Pluye P Ridde V, Dagenais C Les méthodes mixtes pour l'évaluation des programmes Approch Prat En Éval Programme Seconde Édition 2012 Montréal: Presses de l'Université de Montréal 125 143

[B23] Olivier de Sardan J-P Ridde V J.-P. Olivier de Sardan and V. Ridde Introduction. Diagnostic d'une politique publique: les exemptions de paiement pour les soins de santé au Sahel. Une politique publique de santé et ses contradictions. La gratuité des soins au Burkina Faso, au Mali et au Niger 2014 Karthala

[B24] Olivier de Sardan J-P Ridde V J.-P. Olivier de Sardan and V. Ridde Les spécificités des politiques publiques et des systèmes de santé en Afrique sahélienne. Une politique publique de santé et ses contradictions. La gratuité des soins au Burkina Faso, au Mali et au Niger 2014 Karthala

[B25] Olivier de Sardan J-P J.-P. Olivier de Sardan and V. Ridde La quantité sans la qualité? Mises en forme et mises en oeuvre des politiques d'exemptions de paiements au Sahel Une politique publique de santé et ses contradictions. La gratuité des soins au Burkina Faso, au Mali et au Niger 2014 Karthala

